# A Flexible Three-in-One Microsensor for Real-Time Monitoring of Internal Temperature, Voltage and Current of Lithium Batteries

**DOI:** 10.3390/s150511485

**Published:** 2015-05-19

**Authors:** Chi-Yuan Lee, Huan-Chih Peng, Shuo-Jen Lee, I-Ming Hung, Chien-Te Hsieh, Chuan-Sheng Chiou, Yu-Ming Chang, Yen-Pu Huang

**Affiliations:** 1 Department of Mechanical Engineering, Yuan Ze Fuel Cell Center, Yuan Ze University, Taoyuan 32003, Taiwan; E-Mails: s988705@mail.yzu.edu.tw (H.-C.P.); mesjl@saturn.yzu.edu.tw (S.-J.L.); mecschiu@saturn.yzu.edu.tw (C.-S.C.); s1005066@mail.yzu.edu.tw (Y.-M.C.); s1035061@mail.yzu.edu.tw (Y.-P.H.); 2 Department of Chemical Engineering & Materials Science, Yuan Ze University, Taoyuan 32003, Taiwan; E-Mails: imhung@saturn.yzu.edu.tw (I.-M.H.); cthsieh@saturn.yzu.edu.tw (C.-T.H.)

**Keywords:** lithium battery, micro-electro-mechanical systems, flexible three-in-one micro- sensor, internal monitoring

## Abstract

Lithium batteries are widely used in notebook computers, mobile phones, 3C electronic products, and electric vehicles. However, under a high charge/discharge rate, the internal temperature of lithium battery may rise sharply, thus causing safety problems. On the other hand, when the lithium battery is overcharged, the voltage and current may be affected, resulting in battery instability. This study applies the micro-electro-mechanical systems (MEMS) technology on a flexible substrate, and develops a flexible three-in-one microsensor that can withstand the internal harsh environment of a lithium battery and instantly measure the internal temperature, voltage and current of the battery. Then, the internal information can be fed back to the outside in advance for the purpose of safety management without damaging the lithium battery structure. The proposed flexible three-in-one microsensor should prove helpful for the improvement of lithium battery design or material development in the future.

## 1. Introduction 

Many countries are devoted to alleviating global warming and finding coping strategies, especially with the development of green energy. The green energy industry includes wind power, tidal power generation, hydropower and solar power generation. These green energies use pollution-free energy sources to replace the traditional power generation systems which produce greenhouse gases. However, if these power generation systems lack a good energy storage mechanism, the excess energy will be wasted. Therefore, it is required to use energy storage devices to store the excess energy. Lithium batteries are a useful tool for energy storage. 

Lithium batteries are characterized by portability, high energy density, high operating voltage, wide service temperature range, no memory effect and long life. Hence, they are indispensable energy storage devices at present. However, in the lithium battery charging/discharging process, the anode material and electrolyte perform electrochemical reactions, which generate a great deal of heat [1]. The overcharge/overdischarge can result in voltage instability an even thermal runaway [2], as well as safety problems. A new approach, suitable for real-time implementation, was introduced for estimation of the non-uniform internal temperature distribution in cylindrical lithium-ion cells, in which a radial 1-D model is used to estimate the distribution using two inputs: the real or imaginary part of the electrochemical impedance of the cell at a single frequency, and the surface temperature [3]. A preliminary calorimetric analysis and the surface temperatures of high-energy lithium-ion batteries indicated that the cells are prone to thermal runaway at temperatures of approximately 175 ~ 185 °C, which can be triggered by the Joule effect of the short circuit that results from the melting of the separator [4]. Galobardes [5] studied the application of C-MEMS as a lithium-ion battery anode. It is a protective film, referred to as a solid electrolyte interface (SEI), that forms on carbonaceous materials used as negative electrodes in commercial lithium-ion batteries. Chacko [6] studied the electrothermal model of a polymer lithium battery with LiMn_2_O_4_ anode material and graphite cathode material. They also conducted loop tests to draw the battery surface temperature profile models. Wiedemann [7] found that different electrolyte concentrations resulted in different voltage distributions in the lithium battery charge/discharge. Waag [8] presented a lithium battery having a large charge and discharge accelerated aging. Forgez [9] reported measurement of the internal temperature of lithium iron phosphate and coordination with a commercial thermocouple. Internal temperature measurements and surface temperature measurements of LiFePO4/graphite lithium-ion batteries using the model were validated in current-pulse experiments and a complete charge/discharge of the battery and were within 1.5 °C. Lee [10] developed a flexible temperature micro sensor to embed into a lithium battery. Garay [11] used MEMS techniques to develop an interdigitated electrode geometry and a minimum footprint area of 12 mm^2^ for the medical and biological fields. Pomerantseva [12] showed that the internal stresses of battery electrodes during discharge/charge are important for improving the reliability and cycle lifetime of lithium batteries, using the stress evolution observed in a silicon thin-film electrode incorporated into a MEMS device. Ryan [13] demonstrated thin film technologies that could produce a NiOOH cathode layer that was of high quality and only 1–5 microns thick, and demonstrated the feasibility of microscopic batteries for MEMS. Mutyala [14] used a flexible polymer produced on glass substrates and later transferred it onto thin copper foil embedded thin film thermocouples in a lithium ion battery pouch cell for *in-situ* temperature monitoring. Sun [15] reported a thermal model that can qualitatively predict the dynamic cell temperature changes that occur when a lithium ion battery works under adiabatic conditions. Richardson [16] studied a method of estimating the battery cell core and surface temperature using a thermal model coupled with electrical impedance measurements, rather than using direct surface temperature measurements. This proved advantageous compared to previous methods of estimating the temperature from impedance.

Analysis on lithium battery failure is necessary. The endogenous events of lithium batteries can be observed by real-time monitoring of the internal temperature, voltage and current of the battery, as well as by analyzing the electrochemical reactions occurring inside the battery and possible failure causes. The findings of this study can be applied to the improvement of lithium battery materials in the future, and assist lithium battery management systems to monitor the conditions and design safe failure protection early warning systems. 

Existing commercial temperature, voltage and current sensors are unlikely candidates to be embedded in a lithium battery due to their large size. The probable poor airtightness of the packaging may result in electrolyte leakage, influencing the lithium battery performance and safety. The micro-electro-mechanical systems (MEMS) technology is used in this study to develop a flexible three-in-one microsensor which can be embedded in a lithium battery for real-time monitoring of the internal temperature, voltage and current. The proposed design is characterized by good accuracy, high sensitivity and short reaction times, as well as high flexibility and measurement degrees of freedom (DOF). The developed flexible three-in-one microsensor is embedded in a coin cell for real-time monitoring. The reaction inside the lithium battery can be monitored instantly and more accurately by using this method. The internal temperature uniformity and voltage and current variation are analyzed microscopically, completing the measuring tool for internal real-time microscopic monitoring and safety diagnosis of lithium batteries.

## 2. Theory and Design of Microsensors

**Figure 1:**
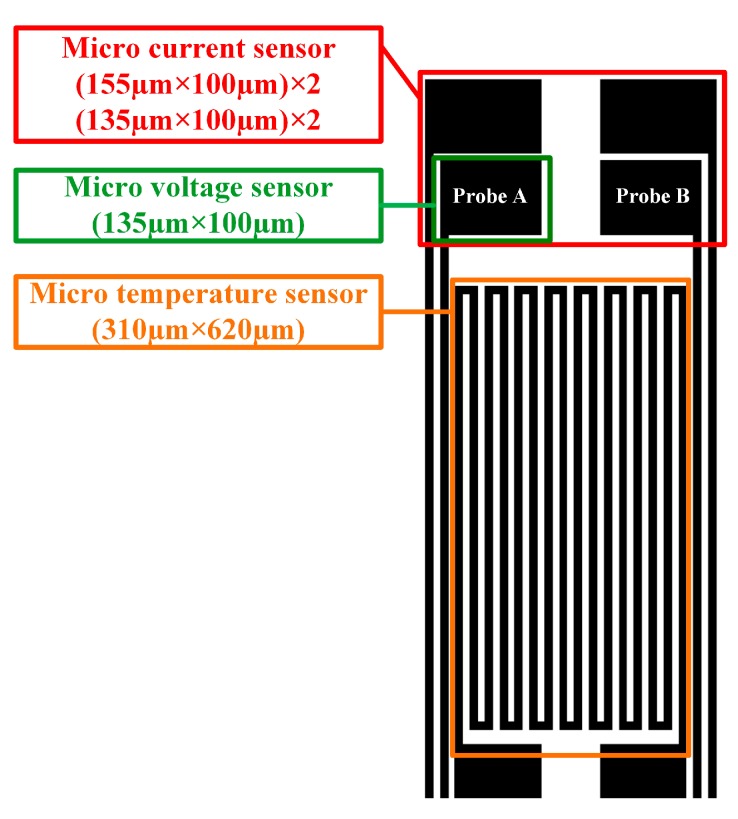
Structural dimensions of the flexible three-in-one microsensor.

The temperature microsensor used in this study was a resistance temperature detector (RTD). The sensed temperature range was wide, and the linearity was good. The serpentine sensing electrode wire of the RTD was 10 μm wide, and the interval was 10 μm. The voltage microsensor was a miniaturized voltmeter probe, and its size was 135 μm × 100 μm. The sensing principle of the current microsensor was that the resistivity (R) of analyte and the voltage difference (V) of analyte were measured. The current value of the analyte was calculated by using Ohm’s law V = I × R. The current microsensor consisted of four miniature probes, including a set of two voltage measuring probes and a set of two resistance measuring probes. Their sizes were 135 μm × 100 μm and 155 μm × 100 μm, respectively. The structure and design of the flexible three-in-one microsensor are shown in Figure 1.

## 3. Fabrication

**Figure 2:**
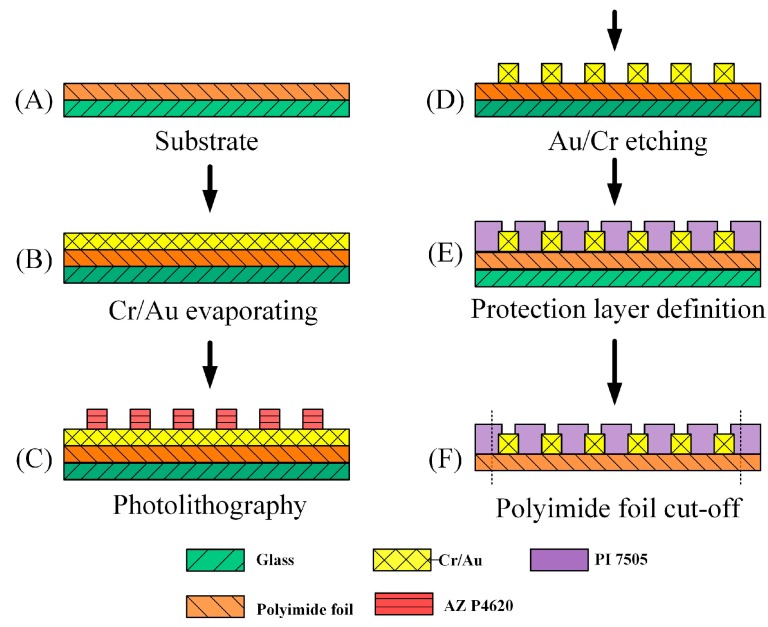
Production process of flexible three-in-one micro sensor.

**Figure 3:**
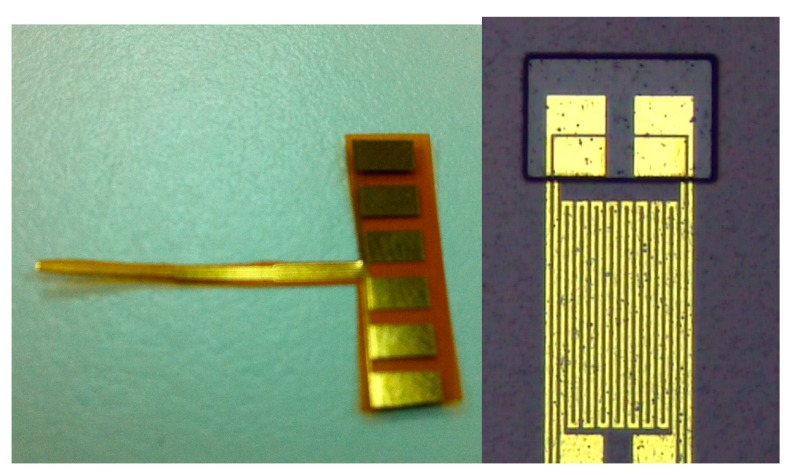
Finished product and optical micrograph of the flexible three-in-one microsensor.

The flexible substrate of this three-in-one microsensor was 50 μm thick polyimide (PI) foil. The foil was cleaned in acetone and methanol. An E-beam evaporator evaporated Cr (500 Å) as adhesion layer and Au (2500 Å) as sensing layer, as shown in Figure 2A,B. The unnecessary Au/Cr film was removed by photolithography with a wet etch to complete the microsensor layout structure, as shown in Figure 2C,D. Finally, polyimide 7505 was spin coated on the sample as insulating layer. The voltage and current probes and sensor pad end were exposed by using a photolithography process again to complete the flexible three-in-one microsensor, as shown in Figure 2E,F. The finished flexible three-in-one microsensor and an optical micrograph are shown in Figure 3.

The coin cell for this test was provided by Professor I-Ming Hung at the Department of Chemical Engineering and Materials Science (Yuan Ze University, Taoyuan, Taiwan). The cathode material was lithium titanium oxide (Li_4_Ti_5_O_12_, LTO). The anode material was lithium iron phosphate (LFP). The lithium battery structure consisted of a top cap, anchor, current collection sheet, cathode electrode, separator, anode electrode, bottom cap and electrolyte. 

**Figure 4:**
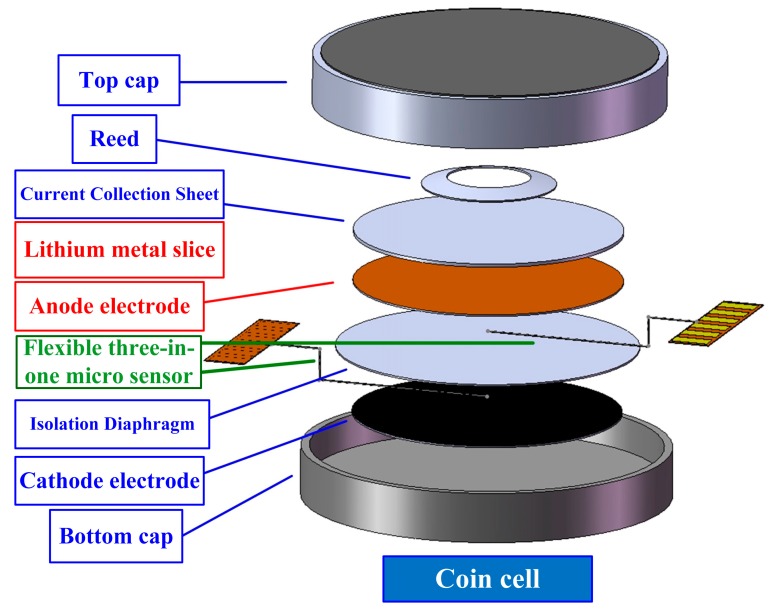
Schematic diagram of the package assembly of the embedded flexible three-in-one microsensors in a coin cell.

The flexible three-in-one microsensors embedded in the lithium battery were numbered sensor 1 and sensor 2. Sensor 1 was embedded between the cathode electrode and separator and facing the cathode electrode. Sensor 2 was embedded between the anode electrode and separator and facing the anode electrode, as shown in Figure 4.

## 4. Coin Cell Test and Internal Real-Time Monitoring

### 4.1. Flexible Three-in-One Microsensor Correction

**Figure 5:**
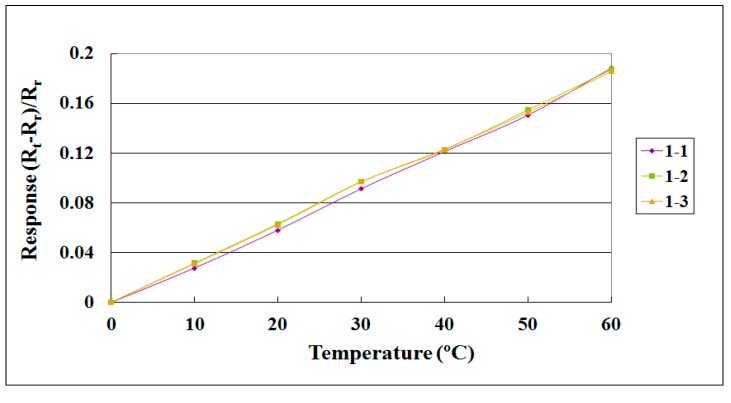
Correction curve of a temperature microsensor (sensor 1).

**Figure 6:**
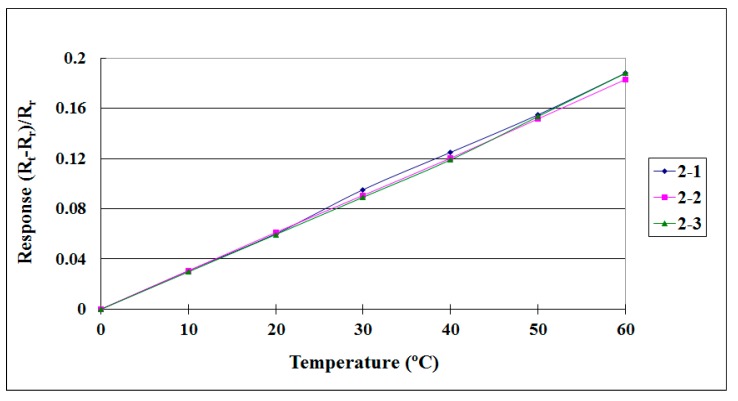
Correction curve of a temperature microsensor (sensor 2).

When the flexible three-in-one microsensor was completed, it was corrected to validate its reliability. After the correction procedure, a lithium battery testing machine and NI data acquisition unit were used for lithium battery tests and internal information acquisition and microscopic diagnostic analysis, to determine the differences in the electric properties of the cells with and without the flexible three-in-one microsensor. The local temperature, voltage and current changes in the lithium battery were monitored and analyzed instantly under different operating conditions. Figure 5 and Figure 6 show the correction curves of two temperature microsensors. Each microsensor showed high linearity and high reproducibility after three correction cycles.

Table 1 shows the voltage correction data of the voltage microsensor. The NI measuring instrument measured the dry battery to obtain the voltage reference. The voltage microsensor then measured the dry battery. The correction difference was obtained by subtracting the voltage measured by the voltage microsensor from the dry battery voltage. It was observed that the voltage error in the voltage measurements resulting from the voltage microsensor conductor was 0.001 V ~ 0.006 V, and the influence was low.

**Table 1:** Correction data of micro voltage sensor.

Sensor	Voltage Correction Difference (mV)		Percentage to Total Voltage (%)	
	Probe A	Probe B	Probe A	Probe B
Sensor 1	9.2998	9.2997	0.000001	0.000003
Sensor 2	9.2996	9.2999	0.000002	0.000001

The current microsensor was corrected by using a standard electrical conductivity solution as reference. The resistivity in the solution was measured by using the current microsensor and converted into electrical conductivity, and compared with the theoretical value of a standard electrical conductivity solution for confirming the reliability of the current microsensor. Table 2 compares the resistivity of the standard electrical conductivity solution measured by the current microsensor with the theoretical value. It was observed that the difference between measured value and theoretical value was less than 1%.

**Table 2:** Comparison between measured value and theoretical value of electrical conductivity.

Sensor	Theoretical Value (µScm^−1^)	Measured Value (µScm^−1^)	Percentage of Difference (%)
Sensor 1	1412	1401	0.78
Sensor 2	1412	1403	0.64

### 4.2. Coin Cell Test

The electrochemical performance of Li-ion batteries was tested using CR2032-type coin cells. The cathode (LiFePO_4_) and anode (Li_4_Ti_5_O_12_) powders were mixed with a binder (polyvinylidene fluoride) and two conducting media (Super-P and KS-4) at a weight ratio of 80:10:5:5 in *N*-methylpyrrolidinone (NMP) solvent to form the electrode slurry. The mixture was blended by a three-dimensional mixer using Zr balls for 3 h to prepare a uniform slurry. Then, the resultant slurry was uniformly pasted on Al (for cathode) and Cu (for anode) foil substrates with a doctor blade, followed by evaporation of the NMP solvent with a blow dryer. The prepared cathode sheets were dried at 135 °C in a vacuum oven for 12 h and pressed under a pressure of approximately 200 kg·cm^−2^. The electrode layers were adjusted to a thickness of ~100 μm. The coin cells were assembled in a glove box for their electrochemical characterization, using an electrochemical analyzer (CHI 608, CH Instruments, Inc., Austin, TX, USA). In the test cells, the Li foil and the porous polypropylene film served as the counter electrode and the separator, respectively. The electrolyte solution was 1.0 M LiPF_6_ in a mixture of ethylene carbonate, polycarbonate, and dimethyl carbonate with a weight ratio of 1:1:1. The charge/discharge cycling tests at different C rates (from 0.1 to 10 C) were performed within the voltage region at ambient temperature.

**Figure 7:**
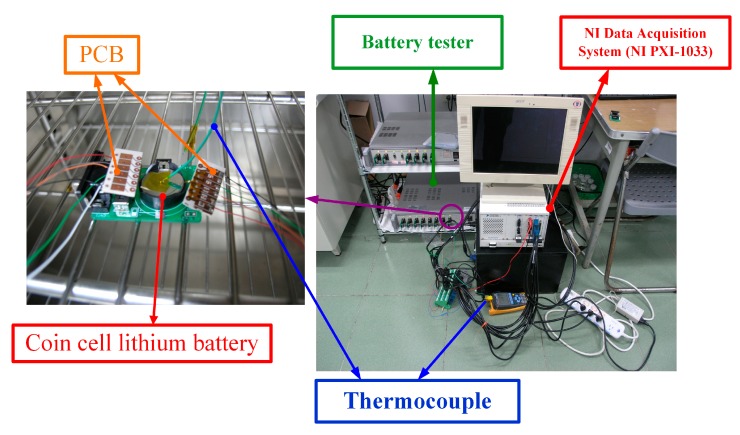
Coin cell test assembly and instrument mounting.

The lithium battery charging/discharging set CHG-5500C was used in this study for testing the coin cell. The coin cell embedded with flexible three-in-one microsensors was placed on the test carrier. A thermocouple temperature recorder was placed on the lithium battery surface to measure and record the battery surface temperature instantly. The NI Data Acquisition System performed real-time measurements and data acquisition of the flexible three-in-one microsensor. Figure 7 shows the coin cell assembly and instrument mounting. The lithium battery was embedded with two flexible three-in-one microsensors.

The anode material of the coin cell was LFP, the cathode material was LTO, and the theoretical capacitance value was 170 mA·h·g^−1^. The test conditions included constant current (CC), charging/discharging voltage range 0.5 ~ 2.9 V. The six charge/discharge rates, ranging from 0.1 to 10 C, are frequently used for evaluating the performance of Li-ion batteries. The performance of Li-ion batteries charged at 0.1–0.5 C reflects the capability for general 3 C portable electronics, whereas the charge-discharge curves at >2 C could serve as a crucial index for evaluating the cell performance for EVs and mobile tools. Nominal capacity (120 mA·h·g^−1^ for LiFePO_4_ cathode), separator (Celgard), maximal charge/discharge rate (10 C), and operating potential range (0.5~3.0 V). The test process is shown in Table 3.

**Table 3:** Coin cell test process (three cycles at each C-rate).

C-Rate	Charge		Discharge	
	Trigger Voltage	Static Voltage	Trigger Voltage	Static Voltage
0.1 C	0.5 V	2.9 V	2.9 V	0.5 V
0.2 C	0.5 V	2.9 V	2.9 V	0.5 V
0.5 C	0.5 V	2.9 V	2.9 V	0.5 V
1 C	0.5 V	2.9 V	2.9 V	0.5 V
5 C	0.5 V	2.9 V	2.9 V	0.5 V
10 C	0.5 V	2.9 V	2.9 V	0.5 V

**Figure 8:**
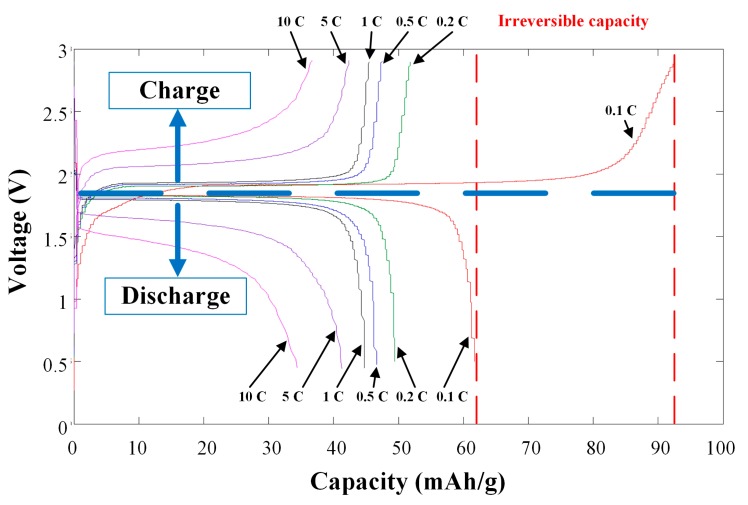
Charge-discharge test curve diagram of the coin cell embedded with flexible three-in-one microsensors.

Figure 8 is the charge-discharge test curve diagram of the coin cell embedded with three-in-one microsensors. The maximum unit cumulative capacity of the 0.1 C charge-discharge test was 92.4731 mA·h·g^−1^, and the maximum unit cumulative capacity of 0.1 C discharge test was 61.7204 mA·h·g^−1^. The calculated irreversible capacity was 30.7527 mA·h·g^−1^, accounting for about 33.26% of the initial value.

**Figure 9:**
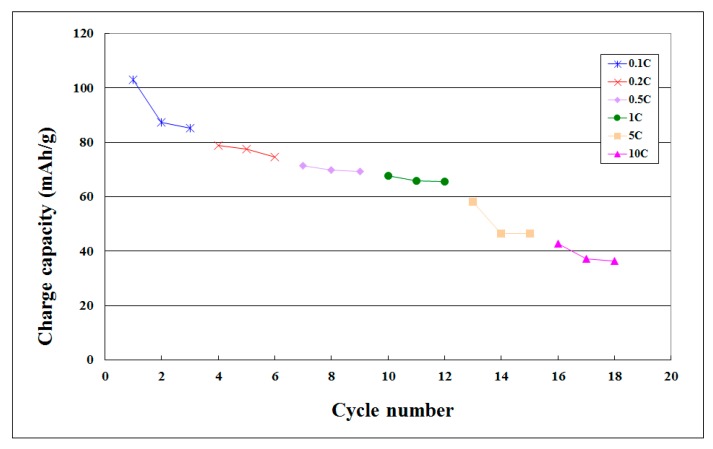
Maximum unit cumulative capacity curve in various cycles of coin cell charge tests.

**Figure 10:**
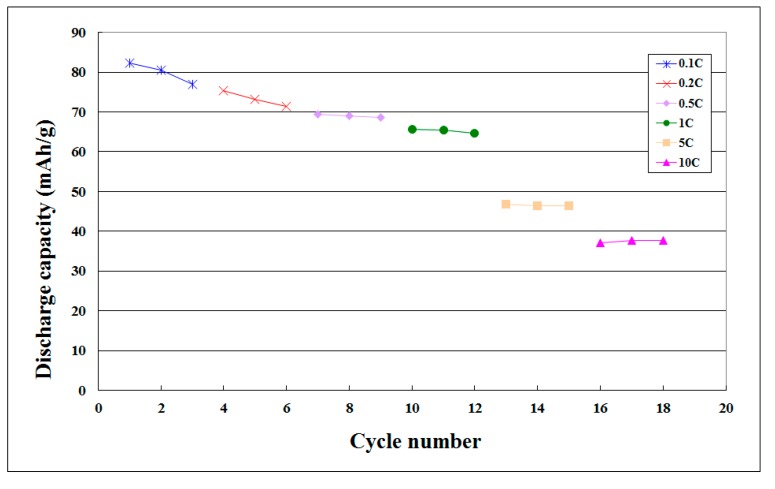
Maximum unit cumulative capacity curve in various cycles of coin cell discharge tests.

Table 4 shows the maximum unit cumulative capacity of charge and discharge of coin cell at various C-rates. Figure 9, Table 5, Figure 10 and Table 6 show the maximum unit cumulative capacity in various cycles of coin cell charge/discharge tests. The electrical performance of the lithium battery in maximum unit cumulative capacity was not good. In the 5 C charge-discharge test, the residual capacity still accounted for 45.81% of the initial value, and even for 39.77% of the initial value in the 10 C charge-discharge test. The lithium battery charge/discharge test did not have complete failure in the end and the lithium battery could complete the overall charge-discharge test process.

**Table 4:** Comparison of maximum unit cumulative capacity of coin cell charge and discharge at various C-rates.

C-Rate	Maximum Unit Cumulative Capacity (mA·h·g^−1^)	
	Charge	Discharge
0.1 C	92.4731	61.7204
0.2 C	51.8280	49.4624
0.5 C	47.5269	46.6667
1 C	45.5914	44.7311
5 C	42.3656	41.2903
10 C	36.7742	34.4086

**Table 5:** Maximum unit cumulative capacity in various cycles of coin cell charge tests.

C-Rate	Cycle Number	Charge Capacity (mAh/g)
0.1 C	1	103.117
	2	82.2727
	3	85.1948
0.2 C	4	78.961
	5	77.4026
	6	74.5455
0.5 C	7	71.4286
	8	69.8701
	9	69.3506
1 C	10	67.7922
	11	65.7143
	12	65.4545
5 C	13	58.1818
	14	46.4935
	15	46.4935
10 C	16	42.8571
	17	37.1429
	18	36.3636

**Table 6:** Maximum unit cumulative capacity in various cycles of coin cell discharge tests.

C-Rate	Cycle Number	Discharge Capacity (mA·h/g)
0.1 C	1	82.3377
	2	80.5195
	3	76.8831
0.2 C	4	75.3247
	5	73.2468
	6	71.4286
0.5 C	7	69.3506
	8	69.0909
	9	68.5714
1 C	10	65.7143
	11	65.4545
	12	64.6753
5 C	13	46.7532
	14	46.4935
	15	46.4935
10 C	16	37.6623
	17	37.6623
	18	37.142

Table 7 shows the performance of the lithium batteries with and without the three-in-one microsensors. The maximum performance difference between the lithium batteries with and without the three-in-one microsensors was only about 10.32%. CA ratio is the weight ratio of anode and cathode materials. In the operation of lithium batteries, the cathode releases lithium ions, and the anode receives the lithium ions. When the releasing capacity of cathode was higher than the receptivity of the anode, the lithium ions could not be received by the anode completely in discharge, so the maximum capacitance value could not be reached. If the releasing capacity of the cathode was lower than the receptivity of the anode, the anode could not release lithium ions completely to the cathode during charge. The lithium battery performance was thus influenced. The difference between CA ratios with and without three-in-one micro sensor was 8.64%. Disregarding this factor, the influence of the three-in-one microsensor on the lithium battery performance was 1.68%. Therefore, the flexible three-in-one microsensor embedded in the lithium battery for real-time measurement had only a slight influence on the electrical performance of the battery. Basically, the specific capacity as a decreasing function of C rate can be attributed to a polarization situation, indicating poor electronic conductivity and slow ionic diffusion rate. On the basis of the experimental results, the degradation of specific capacity at high C rates is minor, e.g., the capacity retention still remains at >60 for the ratio of specific capacity at 0.1 C to 5 C.

**Table 7:** Comparison of performance of lithium batteries with and without embedded three-in-one microsensors.

		With Microsensor	Without Microsensor	Difference Ratio
Maximum cumulative unit capacity (mA·h·g^−1^)	Charge	92.4731	103.1169	10.32%
	Discharge	61.7204	68.5741	9.99%

### 4.3. Persistence Effect Test for the Flexible Three-in-One Microsensors

**Figure 11:**
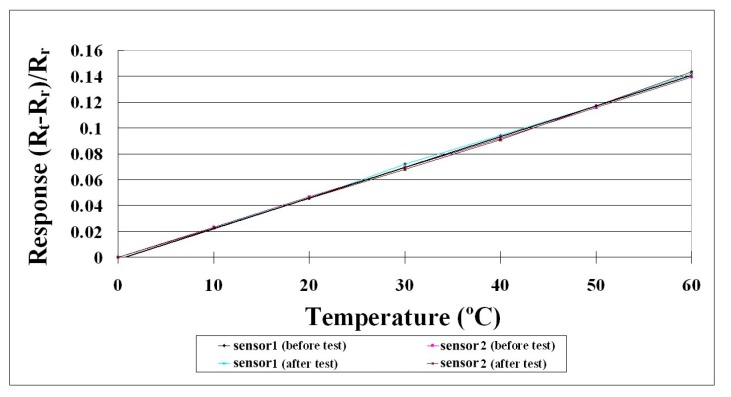
Microsensor temperature correction curve before and after coin cell charge-discharge tests.

The total time for the lithium battery charge/discharge test was about 109.8 h, 0.1 C accounted for 60 h, 0.2 C accounted for 30 h, 0.5 C accounted for 12 h, 1 C accounted for 6 h, 5 C accounted for 1.2 h and 10 C accounted for 0.6 h. The monitoring data are shown in Table 8. After the coin cell charge-discharge test, the flexible three-in-one microsensor performed temperature correction again, as shown in Figure 11. The correction curve still showed high linearity, suggesting that the flexible three-in-one microsensor is durable and reliable.

**Table 8:** Internal monitoring data of the persistence effect test for lithium battery charge/discharge.

	Internal-External Temperature Difference (°C)		Voltage Difference (V)		Current (mA)	
SENSOR No.	1	2	1	2	1	2
0.1 C	0.55	0.56	0.1306	0.0750	0.169	0.170
0.2 C	0.78	0.76	0.1260	0.0714	0.172	0.171
0.5 C	0.85	0.86	0.1301	0.0660	0.184	0.173
1 C	0.97	0.98	0.1312	0.0690	0.186	0.184
5 C	0.60	0.65	0.1276	0.0760	0.193	0.190
10 C	0.45	0.30	0.1351	0.0790	0.199	0.199

## 5. Conclusions

The flexible micro temperature-voltage-current sensor was successfully integrated into PI by using MEMS technology in this study. The total thickness of three-in-one microsensor was 58 μm. It is characterized by quick response, real-time measurement and good durability. 

After the temperature, voltage and electrical conductivity correction of the flexible microsensor, the temperature correction curve shows high linearity and good reproducibility. The voltage and electrical conductivity correction shows the error value of microsensor measurement is smaller than 1%, proving the reliability of the flexible microsensor in temperature, voltage and current measurements. The flexible three-in-one microsensor was successfully embedded in a coin cell in this study. According to the performance of the batteries with and without three-in-one microsensors, the three-in-one micro- sensor could measure the internal temperature, voltage and current of coin cell instantly without disturbing the operation of the lithium battery.
